# Entropy generation and flow characteristics of Powell Eyring fluid under effects of time sale and viscosities parameters

**DOI:** 10.1038/s41598-023-35630-6

**Published:** 2023-05-24

**Authors:** Mohsan Hassan, Muhammad Ahsan, Usman, Metib Alghamdi, Taseer Muhammad

**Affiliations:** 1 Department of Mathematics, COMSATS University Islamabad, Lahore Campus, Lahore, 54000 Pakistan; 2 Department of Computer Science, National University of Sciences and Technology, Balochistan Campus (NBC), Quetta, 87300 Pakistan; 3 Department of Mathematics, College of Sciences, King Khalid University, Abha, 61413 Saudi Arabia

**Keywords:** Mathematics and computing, Physics

## Abstract

Shear thinning fluids are widely used in the food and polymer industries due to their unique flow characteristics. The flow behavior of these fluids has been commonly studied using the Powell Eyring model under a small shear rate assumption. However, this assumption is not always valid. In this study, we explore the transport characteristics of a Powell Eyring fluid over a variable thicker sheet, not only at small shear rates but also at medium and high shear rates. Furthermore, we calculate the rate of entropy generation based on the assumptions. Generalized Powell–Eyring model of viscosity is used for the fluid, representing the re-arrangements of molecules in the forward and backward directions through the theory of potential energy. The model concludes the sensitivity of the viscosity from zero to infinite shear rate along time sale and exponent parameters. The model is used in the transport phenomena equations. The solution of the equation is obtained by using the numerical method and used to calculate the rate of entropy generation. The results are presented in the form of velocity and temperature profiles, the average rate of entropy generation, skin friction coefficient and Nusselt number under the influence of various viscosity parameters. It is found that velocity and temperature profiles are decreased and increased respectively against the time scale parameter.

## Introduction

Fluid flow is a fundamental phenomenon in the chemical process industry, where the flow pattern and the type of liquid/fluid significantly impact the equipment layout, process performance, and economic situation. To efficiently and safely run a chemical plant, it is crucial to anticipate the behavior of fluid flow, along with prior knowledge of heat and mass transfer and boundary conditions. While predicting the behavior of Newtonian liquids, such as water, alcohols, and esters, is relatively straightforward, it becomes much more challenging for non-Newtonian fluids with complex rheological patterns and flow behaviors^1,2^. Shear thinning is a common phenomenon observed in non-Newtonian fluids, wherein the viscosity of the fluid decreases with an increase in shear rate. The viscosity of shear-thinning fluids is a function of shear rate and exhibits Newtonian behavior at very low and high shear rate values. Shear thinning fluids have numerous examples and applications in daily life and the food industry, such as melted chocolate, tomato paste in the ketchup industry, and yoghurt. Polymer melts of high- and low-density polyethene, nylon, polyester, and polystyrene are a few examples of shear-thinning fluids. Other examples of shear-thinning liquids in the cosmetic industry include nail polish, toothpaste, and moisturizing lotions. Given the significant applications of shear-thinning fluids, it is essential to investigate their rheological and flow behaviors and patterns to ensure smooth operation in the chemical process industry. Accurately predicting and understanding the behavior of these fluids can lead to simpler equipment design, avoiding unnecessary complexity throughout the process and ensuring optimal process performance and safety.

There exist numerous mathematical models in the literature to describe the rheological behavior of shear-thinning fluids. The first model proposed by Ostwald in 1929 established a simple relation between shear stress and shear rate^3^. This model, commonly known as the “power law”, became the basis for various viscometers and rheometers used to gather rheological data for different shear-thinning fluids. However, the power law is not suitable for predicting the behavior of shear-thinning fluids at low and high shear rate values. In 1965, Cross proposed another mathematical expression to describe the rheological behavior of inelastic fluids, which is comparatively better than the power law in terms of predicting the rheological properties of shear-thinning fluids at low and high shear rate values^4^. Carreau introduced a model in 1972 based on the molecular structure of shear-thinning fluids^5^. By considering the molecular structure, it is possible to predict the rheological behavior of these fluids by establishing a relationship between viscosity and shear rate. Another general relation between viscosity and shear rate, in the form of an inverse hyperbolic sine, is presented in references^6,7^ and defined as

1 $$\mu = \mu_{\infty } + \left( {\mu_{o} - \mu_{\infty } } \right)\left( {\frac{{\sinh^{ - 1} \left( {\lambda \left| {\overline{\dot{\gamma }}} \right|} \right)}}{{\lambda \left| {\overline{\dot{\gamma }}} \right|}}} \right)^{\sigma } .$$

where $\mu_{o}$ and $\mu_{\infty }$ are viscosities at low and high shear rate respectively, $\overline{\dot{\gamma }}$ is shear rate, $\lambda$ is time sale parameter and $\sigma$ is exponent parameter. The Sutterby model is attained when $\mu_{\infty } = 0$ and The Eyring model is gotten when $\mu_{\infty } = 0$ and $\sigma = 1$. The Eyring–Powell model is represented in Eq. (1) when $\sigma = 1$. Equation (1) is extensively used to predict the flow patterns of shear thinning non-Newtonian fluids for different applications as follows in Table 1.

**Table 1:** The non-Newtonian Eyring–Powell model over diverse geometries^8–20^.

Authors	Model	Geometry	Remarks/investigations
Arshad et al.^8^	$$\sigma = 1$$, $$\mu_{\infty } = 0$$, $$\lambda \left| {\overline{\dot{\gamma }}} \right| \ll 1$$$$\sinh^{ - 1} \left( {\lambda \left| {\overline{\dot{\gamma }}} \right|} \right) \approx \lambda \left| {\overline{\dot{\gamma }}} \right| - \frac{1}{6}\left( {\lambda \left| {\overline{\dot{\gamma }}} \right|} \right)^{3}$$	Peristaltic parallel walls	Thermal behavior of hybrid nanomaterial in fluid flow
Mubbashar Nazeer et al.^9^	$$\sigma = 1$$, $$\mu_{\infty } = 0$$, $$\lambda \left| {\overline{\dot{\gamma }}} \right| \ll 1$$$$\sinh^{ - 1} \left( {\lambda \left| {\overline{\dot{\gamma }}} \right|} \right) \approx \lambda \left| {\overline{\dot{\gamma }}} \right| - \frac{1}{6}\left( {\lambda \left| {\overline{\dot{\gamma }}} \right|} \right)^{3}$$	Parallel walls	Comparative study of Crossflow of heat and mass
Oke et al.^10^	$$\sigma > 1,$$$$\mu_{\infty } = 0$$, $$\lambda \left| {\overline{\dot{\gamma }}} \right| \ll 1$$$$\sinh^{ - 1} \left( {\lambda \left| {\overline{\dot{\gamma }}} \right|} \right) \approx \lambda \left| {\overline{\dot{\gamma }}} \right| - \frac{1}{6}\left( {\lambda \left| {\overline{\dot{\gamma }}} \right|} \right)^{3}$$	Linear stretching sheet	Shear thinning and thickening effects of heat and mass flow
Rahimi et al.^11^	$$\sigma = 1$$, $$\mu_{\infty } = 0$$, $$\lambda \left| {\overline{\dot{\gamma }}} \right| \ll 1$$$$\sinh^{ - 1} \left( {\lambda \left| {\overline{\dot{\gamma }}} \right|} \right) \approx \lambda \left| {\overline{\dot{\gamma }}} \right| - \frac{1}{6}\left( {\lambda \left| {\overline{\dot{\gamma }}} \right|} \right)^{3}$$	Linear stretching sheet	Solution by collocation method of boundary layer fluid flow
Verma et al.^12^	$$\sigma = 1$$, $$\mu_{\infty } = 0$$, $$\lambda \left| {\overline{\dot{\gamma }}} \right| \ll 1$$$$\sinh^{ - 1} \left( {\lambda \left| {\overline{\dot{\gamma }}} \right|} \right) \approx \lambda \left| {\overline{\dot{\gamma }}} \right| - \frac{1}{6}\left( {\lambda \left| {\overline{\dot{\gamma }}} \right|} \right)^{3}$$	Moving plate	Stability analysis and multiple solutions of nanofluid flow
Ali Akgul^13^	$$\sigma = 1$$, $$\mu_{\infty } = 0$$, $$\lambda \left| {\overline{\dot{\gamma }}} \right| \ll 1$$$$\sinh^{ - 1} \left( {\lambda \left| {\overline{\dot{\gamma }}} \right|} \right) \approx \lambda \left| {\overline{\dot{\gamma }}} \right| - \frac{1}{6}\left( {\lambda \left| {\overline{\dot{\gamma }}} \right|} \right)^{3}$$	Linear stretching sheet	Solution of boundary layer flow by Hilbert space method
Imran et al.^14^	$$\sigma = 1$$, $$\mu_{\infty } = 0$$, $$\lambda \left| {\overline{\dot{\gamma }}} \right| \ll 1$$$$\sinh^{ - 1} \left( {\lambda \left| {\overline{\dot{\gamma }}} \right|} \right) \approx \lambda \left| {\overline{\dot{\gamma }}} \right| - \frac{1}{6}\left( {\lambda \left| {\overline{\dot{\gamma }}} \right|} \right)^{3}$$	Oscillatory curved sheet	Heat transfer analysis through Fourier and Fick model
Ibrahim and Hindebu^15^	$$\sigma = 1$$, $$\mu_{\infty } = 0$$, $$\lambda \left| {\overline{\dot{\gamma }}} \right| \ll 1$$$$\sinh^{ - 1} \left( {\lambda \left| {\overline{\dot{\gamma }}} \right|} \right) \approx \lambda \left| {\overline{\dot{\gamma }}} \right| - \frac{1}{6}\left( {\lambda \left| {\overline{\dot{\gamma }}} \right|} \right)^{3}$$	Stretching cylinder	Heat transfer analysis in nanofluid through the Cattaneo-Christov model
Kumar et al.^16^	$$\sigma = 1$$, $$\mu_{\infty } = 0$$, $$\lambda \left| {\overline{\dot{\gamma }}} \right| \ll 1$$$$\sinh^{ - 1} \left( {\lambda \left| {\overline{\dot{\gamma }}} \right|} \right) \approx \lambda \left| {\overline{\dot{\gamma }}} \right| - \frac{1}{6}\left( {\lambda \left| {\overline{\dot{\gamma }}} \right|} \right)^{3}$$	Annulus	Magnetic and rotation effects on mass flow
Gholinia et al.^17^	$$\sigma = 1$$, $$\mu_{\infty } = 0$$, $$\lambda \left| {\overline{\dot{\gamma }}} \right| \ll 1$$$$\sinh^{ - 1} \left( {\lambda \left| {\overline{\dot{\gamma }}} \right|} \right) \approx \lambda \left| {\overline{\dot{\gamma }}} \right| - \frac{1}{6}\left( {\lambda \left| {\overline{\dot{\gamma }}} \right|} \right)^{3}$$	Rotating disk	Homogeneous–heterogeneous reactions on heat and mass flow
Salawu et al.^18^	$$\sigma = 1$$, $$\mu_{\infty } = 0$$, $$\lambda \left| {\overline{\dot{\gamma }}} \right| \ll 1$$$$\sinh^{ - 1} \left( {\lambda \left| {\overline{\dot{\gamma }}} \right|} \right) \approx \lambda \left| {\overline{\dot{\gamma }}} \right| - \frac{1}{6}\left( {\lambda \left| {\overline{\dot{\gamma }}} \right|} \right)^{3}$$	Channel	Entropy generation of fluid under magnetic effects
Madhu et al.^19^	$$\sigma = 1$$, $$\mu_{\infty } = 0$$, $$\lambda \left| {\overline{\dot{\gamma }}} \right| \ll 1$$$$\sinh^{ - 1} \left( {\lambda \left| {\overline{\dot{\gamma }}} \right|} \right) \approx \lambda \left| {\overline{\dot{\gamma }}} \right| - \frac{1}{6}\left( {\lambda \left| {\overline{\dot{\gamma }}} \right|} \right)^{3}$$	Microchannel	Heat and mass transfer in the presence of a thermal heat source
Çolak et al.^20^	$$\sigma = 1$$, $$\mu_{\infty } = 0$$, $$\lambda \left| {\overline{\dot{\gamma }}} \right| \ll 1$$$$\sinh^{ - 1} \left( {\lambda \left| {\overline{\dot{\gamma }}} \right|} \right) \approx \lambda \left| {\overline{\dot{\gamma }}} \right| - \frac{1}{6}\left( {\lambda \left| {\overline{\dot{\gamma }}} \right|} \right)^{3}$$	Stretching sheet	Bioconvective in nanofluid

The studies mentioned above indicate that Eq. (1) has been extensively examined in fluid flow problems across various geometries, subject to certain assumptions $\sigma = 1$, $\mu_{\infty } = 0$ and $\lambda \left| {\overline{\dot{\gamma }}} \right| \ll 1.$ The viscosity of some shear-thinning liquids can be altered by a factor of three to four relative to the shear rate, making it impossible to disregard such a substantial change in viscosity during the processing of polymer melts or lubes. Consequently, the aforementioned assumptions cannot be considered universally applicable. The present problem's objective is to investigate Eyring–Powell fluid flow with heat transfer while taking into account viscosity at low shear rates, which has not been previously addressed. The flow is analyzed over a variable nonlinear thicker stretching sheet, with boundary layer assumptions applied due to the proximity of the flow to the wall. The problem consists of highly nonlinear differential equations that are solved using numerical techniques. The results are expressed as velocity and temperature equations, which are then used to calculate physical quantities.

## Mathematical model

**Figure 1:**
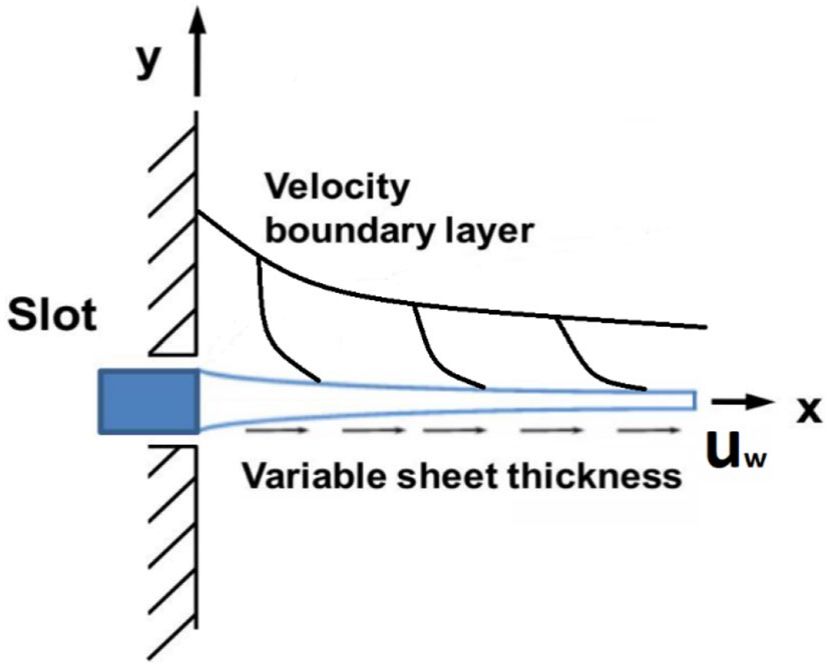
The flow geometry for the problem.

Consider the incompressible, steady-state and laminar boundary layer flow of the non-Newtonian fluid through the Generalized Powell–Eyring model over a variable thicker sheet. The flow geometry is shown in Fig. 1.

The sheet is subjected to stretching at a variable velocity, denoted by a function $u_{w} = a\left( {\overline{x} + b} \right)^{m}$. Additionally, the thickness of the sheet varies with a specific function $y = A\left( {\overline{x} + b} \right)^{{\frac{1 - m}{2}}}$. It is assumed that the temperature of the sheet remains constant $T_{w}$ and is greater than the temperature of the inviscid region denoted by $T_{\infty }$.

The mathematical flow model is based on partial differential equations that represent continuity, momentum, and energy equations as follows

2 $$\overline{u}_{{\overline{x}}} + \overline{v}_{{\overline{y}}} = 0,$$

3 $$\rho \left( {\overline{u}\overline{u}_{{\overline{x}}} + \overline{v}\overline{u}_{{\overline{y}}} } \right) = - \overline{p}_{{\overline{x}}} + \left( {\overline{\tau }_{11} } \right)_{{\overline{x}}} + \left( {\overline{\tau }_{12} } \right)_{{\overline{y}}} ,$$

4 $$\rho \left( {\overline{u}\overline{v}_{{\overline{x}}} + \overline{v}\overline{v}_{{\overline{y}}} } \right) = - \overline{p}_{{\overline{y}}} + \left( {\overline{\tau }_{21} } \right)_{{\overline{x}}} + \left( {\overline{\tau }_{22} } \right)_{{\overline{y}}} ,$$

5 $$\rho C_{p} \left( {\overline{u}\overline{T}_{{\overline{x}}} + \overline{v}\overline{T}_{{\overline{y}}} } \right) = k\left( {\overline{T}_{{\overline{x}\overline{x}}} + \overline{T}_{{\overline{y}\overline{y}}} } \right) + \overline{\phi }.$$

In the above, the shear stresses $\tau_{ij}$ for the Generalized Powell–Eyring model are defined as

6 $$\begin{aligned} \overline{\tau }_{11} & = \mu_{\infty } + \left( {\mu_{o} - \mu_{\infty } } \right)\left( {\frac{{\sinh^{ - 1} \left( {\lambda \left| {\overline{\dot{\gamma }}} \right|} \right)}}{{\lambda \left| {\overline{\dot{\gamma }}} \right|}}} \right)^{\sigma } \left( {2\overline{u}_{{\overline{x}}} } \right) \\ \overline{\tau }_{12} & = \overline{\tau }_{21} = \mu_{\infty } + \left( {\mu_{o} - \mu_{\infty } } \right)\left( {\frac{{\sinh^{ - 1} \left( {\lambda \left| {\overline{\dot{\gamma }}} \right|} \right)}}{{\lambda \left| {\overline{\dot{\gamma }}} \right|}}} \right)^{\sigma } \left( {\overline{u}_{{\overline{y}}} + \overline{v}_{{\overline{x}}} } \right) \\ \overline{\tau }_{22} & = \mu_{\infty } + \left( {\mu_{o} - \mu_{\infty } } \right)\left( {\frac{{\sinh^{ - 1} \left( {\lambda \left| {\overline{\dot{\gamma }}} \right|} \right)}}{{\lambda \left| {\overline{\dot{\gamma }}} \right|}}} \right)^{\sigma } \left( {2\overline{v}_{{\overline{y}}} } \right) \\ \end{aligned}$$

and viscous dissipation $\overline{\phi }$ is written as

7 $$\overline{\phi } = \left\{ {\mu_{\infty } + \left( {\mu_{o} - \mu_{\infty } } \right)\left( {\frac{{\sinh^{ - 1} \left( {\lambda \left| {\overline{\dot{\gamma }}} \right|} \right)}}{{\lambda \left| {\overline{\dot{\gamma }}} \right|}}} \right)^{\sigma } } \right\}\left( {2\overline{v}_{{\overline{y}}}^{2} + \left( {\overline{u}_{{\overline{y}}} + \overline{v}_{{\overline{x}}} } \right)^{2} + 2\overline{u}_{{\overline{x}}}^{2} } \right).$$

In view of Eqs. (5) and (6), Eqs. (2)–(4) are written as

8 $$\begin{aligned} \rho \left( {\overline{u}\overline{u}_{{\overline{x}}} + \overline{v}\overline{u}_{{\overline{y}}} } \right) & = - \overline{p}_{{\overline{x}}} + \left( {\mu_{\infty } + \left( {\mu_{o} - \mu_{\infty } } \right)\left( {\frac{{\sinh^{ - 1} \left( {\lambda \left| {\overline{\dot{\gamma }}} \right|} \right)}}{{\lambda \left| {\overline{\dot{\gamma }}} \right|}}} \right)^{\sigma } \left( {2\overline{u}_{{\overline{x}}} } \right)} \right)_{{\overline{x}}} \\ & \quad + \left( {\mu_{\infty } + \left( {\mu_{o} - \mu_{\infty } } \right)\left( {\frac{{\sinh^{ - 1} \left( {\lambda \left| {\overline{\dot{\gamma }}} \right|} \right)}}{{\lambda \left| {\overline{\dot{\gamma }}} \right|}}} \right)^{\sigma } \left( {\overline{u}_{{\overline{y}}} + \overline{v}_{{\overline{x}}} } \right)} \right)_{{\overline{y}}} , \\ \end{aligned}$$

9 $$\begin{aligned} \rho \left( {\overline{u}\overline{v}_{{\overline{x}}} + \overline{v}\overline{v}_{{\overline{y}}} } \right) & = - \overline{p}_{{\overline{y}}} + \left( {\mu_{\infty } + \left( {\mu_{o} - \mu_{\infty } } \right)\left( {\frac{{\sinh^{ - 1} \left( {\lambda \left| {\overline{\dot{\gamma }}} \right|} \right)}}{{\lambda \left| {\overline{\dot{\gamma }}} \right|}}} \right)^{\sigma } \left( {\overline{u}_{{\overline{y}}} + \overline{v}_{{\overline{x}}} } \right)} \right)_{{\overline{x}}} \\ & \quad + \left( {\mu_{\infty } + \left( {\mu_{o} - \mu_{\infty } } \right)\left( {\frac{{\sinh^{ - 1} \left( {\lambda \left| {\overline{\dot{\gamma }}} \right|} \right)}}{{\lambda \left| {\overline{\dot{\gamma }}} \right|}}} \right)^{\sigma } \left( {2\overline{v}_{{\overline{y}}} } \right)} \right)_{{\overline{y}}} , \\ \end{aligned}$$

10 $$\begin{aligned} \rho C_{p} \left( {\overline{u}\overline{T}_{{\overline{x}}} + \overline{v}\overline{T}_{{\overline{y}}} } \right) & = k\left( {\overline{T}_{{\overline{x}\overline{x}}} + \overline{T}_{{\overline{y}\overline{y}}} } \right) \\ & \quad + \left\{ {\mu_{\infty } + (\mu_{o} - \mu_{\infty } )\left( {\frac{{\sinh^{ - 1} \left( {\lambda \left| {\overline{\dot{\gamma }}} \right|} \right)}}{{\lambda \left| {\overline{\dot{\gamma }}} \right|}}} \right)^{\sigma } } \right\}\left\{ {2\overline{v}_{{\overline{y}}}^{2} + \left( {\overline{u}_{{\overline{y}}} + \overline{v}_{{\overline{x}}} } \right)^{2} + 2\overline{u}_{{\overline{x}}}^{2} } \right\}. \\ \end{aligned}$$

Now, the Eqs. (8)–(10) are reduced through boundary layer assumptions. Consider the following boundary layer assumptions as

11 $$\overline{x} = Lx,\;\overline{y} = \varepsilon Ly,\;\overline{u} = U_{\infty } u,\;\overline{v} = \varepsilon U_{\infty } v,\;\overline{p} = \rho U_{\infty }^{2} p,\;T = \frac{{\overline{T} - T_{\infty } }}{{T_{w} - T_{\infty } }}.$$

Equations (8)–(10) after applying Eq. (11) are written as

12 $$\begin{aligned} \left( {uu_{x} + vu_{y} } \right) & = - p_{x} \\ & \quad + {\text{Re}}^{ - 1} \left[ {\left\{ {\beta + (1 - \beta )\left( {\frac{{\sinh^{ - 1} \left( {\frac{{{\text{Re}}^{ - 1} }}{\varepsilon }C\sqrt {2\varepsilon^{2} v_{y}^{2} + \left( {u_{y} + \varepsilon^{2} v_{x} } \right)^{2} + 2\varepsilon^{2} u_{x}^{2} } } \right)}}{{\left( {\frac{{{\text{Re}}^{ - 1} }}{\varepsilon }C\sqrt {2\varepsilon^{2} v_{y}^{2} + \left( {u_{y} + \varepsilon^{2} v_{x} } \right)^{2} + 2\varepsilon^{2} u_{x}^{2} } } \right)}}} \right)^{\sigma } } \right\}\left( {2u_{x} } \right)} \right]_{x} \\ & \quad + \frac{{{\text{Re}}^{ - 1} }}{{\varepsilon^{2} }}\left[ {\left\{ {\beta + (1 - \beta )\left( {\frac{{\sinh^{ - 1} \left( {\frac{{{\text{Re}}^{ - 1} }}{\varepsilon }C\sqrt {2\varepsilon^{2} v_{y}^{2} + \left( {u_{y} + \varepsilon^{2} v_{x} } \right)^{2} + 2\varepsilon^{2} u_{x}^{2} } } \right)}}{{\left( {\frac{{{\text{Re}}^{ - 1} }}{\varepsilon }C\sqrt {2\varepsilon^{2} v_{y}^{2} + \left( {u_{y} + \varepsilon^{2} v_{x} } \right)^{2} + 2\varepsilon^{2} u_{x}^{2} } } \right)}}} \right)^{\sigma } } \right\}\left( {u_{y} + \varepsilon^{2} v_{x} } \right)} \right]_{y} , \\ \end{aligned}$$

13 $$\begin{aligned} \varepsilon^{2} \left( {uv_{x} + vv_{y} } \right) & = - p_{y} \\ & \quad + {\text{Re}}^{ - 1} \left[ {\left\{ {\beta + (1 - \beta )\left( {\frac{{\sinh^{ - 1} \left( {\frac{{{\text{Re}}^{ - 1} }}{\varepsilon }C\sqrt {2\varepsilon^{2} v_{y}^{2} + \left( {u_{y} + \varepsilon^{2} v_{x} } \right)^{2} + 2\varepsilon^{2} u_{x}^{2} } } \right)}}{{\left( {\frac{{{\text{Re}}^{ - 1} }}{\varepsilon }C\sqrt {2\varepsilon^{2} v_{y}^{2} + \left( {u_{y} + \varepsilon^{2} v_{x} } \right)^{2} + 2\varepsilon^{2} u_{x}^{2} } } \right)}}} \right)^{\sigma } } \right\}\left( {u_{y} + \varepsilon^{2} v_{x} } \right)} \right]_{x} \\ & \quad + {\text{Re}}^{ - 1} \left[ {\left\{ {\beta + (1 - \beta )\left( {\frac{{\sinh^{ - 1} \left( {\frac{{{\text{Re}}^{ - 1} }}{\varepsilon }C\sqrt {2\varepsilon^{2} v_{y}^{2} + \left( {u_{y} + \varepsilon^{2} v_{x} } \right)^{2} + 2\varepsilon^{2} u_{x}^{2} } } \right)}}{{\left( {\frac{{{\text{Re}}^{ - 1} }}{\varepsilon }C\sqrt {2\varepsilon^{2} v_{y}^{2} + \left( {u_{y} + \varepsilon^{2} v_{x} } \right)^{2} + 2\varepsilon^{2} u_{x}^{2} } } \right)}}} \right)^{\sigma } } \right\}\left( {2v_{y} } \right)} \right]_{y} , \\ \end{aligned}$$

14 $$\begin{aligned} \left( {uT_{x} + vT_{y} } \right) & = \Pr^{ - 1} \left( {\frac{{{\text{Re}}^{ - 1} }}{{\varepsilon^{2} }}} \right)\left( {T_{yy} + \varepsilon^{2} T_{xx} } \right) \\ & \quad + Ec\frac{{{\text{Re}}^{ - 1} }}{{\varepsilon^{2} }}\left[ {\beta + (1 - \beta )\left( {\frac{{\sinh^{ - 1} \left( {C\frac{{{\text{Re}}^{ - 1} }}{\varepsilon }\sqrt {2\varepsilon^{2} v_{y}^{2} + \left( {u_{y} + \varepsilon^{2} v_{x} } \right)^{2} + 2\varepsilon^{2} u_{x}^{2} } } \right)}}{{C\frac{{{\text{Re}}^{ - 1} }}{\varepsilon }\sqrt {2\varepsilon^{2} v_{y}^{2} + \left( {u_{y} + \varepsilon^{2} v_{x} } \right)^{2} + 2\varepsilon^{2} u_{x}^{2} } }}} \right)^{\sigma } } \right]\left\{ \begin{gathered} 2\varepsilon^{2} v_{y}^{2} + 2\varepsilon^{2} u_{x}^{2} \hfill \\ \left( {u_{y} + \varepsilon^{2} v_{x} } \right)^{2} \hfill \\ \end{gathered} \right\}. \\ \end{aligned}$$

Here $$Ec = \frac{{U_{\infty }^{2} }}{{C_{p} \left( {T_{w} - T_{\infty } } \right)}}$$, $$\Pr^{ - 1} = \frac{\alpha }{v}$$, $${\text{Re}}^{ - 1} = \frac{{\mu_{o} }}{{U_{\infty } \rho L}}$$ and $$C = \sqrt {\frac{{\mu_{\infty }^{3} \rho \lambda^{2} }}{{\mu_{o} L}}}$$.

For Re >> 1 and

15 $$\frac{{{\text{Re}}^{ - 1} }}{{\varepsilon^{2} }} \approx O\left( 1 \right)\;{\text{and}}\;{\text{Re}}^{ - 1/2} = O\left( \varepsilon \right),$$

the Eqs. (12)–(14) are written without bar are written as

16 $$u_{x} + v_{y} = 0,$$

17 $$\rho \left( {uu_{x} + vu_{y} } \right) = - p_{x} + \left\{ {\mu_{\infty } + \left( {\mu_{ \circ } - \mu_{\infty } } \right)\left( {\frac{{\sinh^{ - 1} \left( {\lambda \sqrt {u_{y}^{2} } } \right)}}{{\lambda \sqrt {u_{y}^{2} } }}} \right)^{\sigma } u_{y} } \right\}_{y} ,$$

18 $$p_{y} = 0,$$

19 $$\rho C_{p} \left( {uT_{x} + vT_{y} } \right) = kT_{yy} + \left\{ {\mu_{\infty } + \left( {\mu_{ \circ } - \mu_{\infty } } \right)\left( {\frac{{\sinh^{ - 1} \left( {\lambda \sqrt {u_{y}^{2} } } \right)}}{{\lambda \sqrt {u_{y}^{2} } }}} \right)^{\sigma } } \right\}u_{y}^{2}$$

along associated boundary conditions

20 $$\begin{aligned} & u\left( {x,A(x + b)^{{\frac{1 - m}{2}}} } \right) = u_{w} ,\;v\left( {x,A(x + b)^{{\frac{1 - m}{2}}} } \right) = 0,\;T\left( {x,A(x + b)^{{\frac{1 - m}{2}}} } \right) = T_{w} \\ & u\left( {x,y \to \infty } \right) \to 0,\;T\left( {x,y \to \infty } \right) \to T_{\infty } . \\ \end{aligned}$$

Now, introduces the similarity transformations to reduce the governing equations into ordinary differential equations as^21^

21 $$\Psi = \sqrt {\nu u_{w} \left( {x + b} \right)} F,\;\eta = y\sqrt {\frac{{u_{w} }}{{\nu \left( {x + b} \right)}}} ,\;\theta \left( \eta \right) = \frac{{T - T_{\infty } }}{{\left( {T_{w} - T_{\infty } } \right)}}.$$

In above $\Psi$ is a stream function and satisfies Eq. (21) by

22 $$u = \frac{\partial \Psi }{{\partial y}},\;v = - \frac{\partial \Psi }{{\partial x}}.$$

In view of Eqs. (21) and (22), the main Eqs. (16)–(20) are simplified as

23 $$\begin{aligned} mF^{\prime 2} - \frac{{\left( {m + 1} \right)}}{2}FF^{\prime \prime } & = \sigma (1 - \beta )\left( {\frac{{\sinh^{ - 1} \left( {C_{x} \sqrt {F^{\prime \prime 2} } } \right)}}{{C_{x} \sqrt {F^{\prime \prime 2} } }}} \right)^{\sigma - 1} \left( { - \frac{{\sinh^{ - 1} \left( {C_{x} \sqrt {F^{\prime \prime 2} } } \right)}}{{C_{x} \sqrt {F^{\prime \prime 2} } }} + \frac{1}{{\sqrt {1 + C_{x}^{2} F^{\prime \prime 2} } }}} \right)F^{\prime \prime \prime } \\ & \quad + \left( {\beta + (1 - \beta )\frac{{\sinh^{ - 1} \left( {C_{x} \sqrt {F^{\prime \prime 2} } } \right)}}{{\left( {C_{x} \sqrt {F^{\prime \prime 2} } } \right)}}} \right)^{\sigma } F^{\prime \prime \prime } , \\ \end{aligned}$$

24 $$- \frac{m + 1}{2}\Pr F\theta^{\prime } = \theta^{\prime \prime } + \Pr Ec\left\{ {\beta + (1 - \beta )\left( {\frac{{\sinh^{ - 1} \left( {C_{x} \sqrt {F^{\prime \prime 2} } } \right)}}{{C_{x} \sqrt {F^{\prime \prime 2} } }}} \right)^{\sigma } } \right\}F^{\prime \prime 2} ,$$

25 $$\begin{array}{*{20}l} {f^{\prime } = 1,\;f = \alpha ,\;\theta = 1} \hfill & {\quad {\text{at}}\;\eta = B} \hfill \\ {f^{\prime } \to 0,\;\theta \to 0} \hfill & {\quad {\text{at}}\;\eta = \infty } \hfill \\ \end{array} ,$$

where $\beta = \frac{{\mu_{\infty } }}{{\mu_{ \circ } }}$, $C_{x} = \lambda \sqrt {\frac{{a^{3} \left( {x + b} \right)^{3m - 1} }}{\nu }}$, $\alpha = - B\left( {\frac{m - 1}{{1 + m}}} \right)$ and $B = A\sqrt {\frac{a}{\nu }}$ are constants.

## Entropy generation

The rate of entropy generation is defined as

26 $$\dot{S}_{gen} = \frac{k}{{T_{\infty }^{2} }}\left( {\nabla T} \right)^{2} + \frac{\mu }{{T_{\infty } }}\overline{\dot{\gamma }}^{2} ,$$

where

27 $$\left( {\nabla T} \right)^{2} = \overline{T}^{2}_{{\overline{x}}} + \overline{T}^{2}_{{\overline{y}}} .$$

By using Eq. (27) into Eq. (26), we get

28 $$\dot{S}_{gen} = \frac{k}{{T_{\infty }^{2} }}\left( {\overline{T}^{2}_{{\overline{x}}} + \overline{T}^{2}_{{\overline{y}}} } \right) + \frac{1}{{T_{\infty } }}\left\{ {\mu_{\infty } + (\mu_{ \circ } - \mu_{\infty } )\left( {\frac{{\sinh^{ - 1} \left( {\lambda \left| {\overline{\dot{\gamma }}} \right|} \right)}}{{\lambda \left| {\overline{\dot{\gamma }}} \right|}}} \right)^{\sigma } } \right\}\left( {2\overline{v}_{{\overline{y}}}^{2} + \left( {\overline{u}_{{\overline{y}}} + \overline{v}_{{\overline{x}}} } \right)^{2} + 2\overline{u}_{{\overline{x}}}^{2} } \right).$$

After applying Eq. (11), we get

29 $$\begin{aligned} \dot{N} & = \frac{{{\text{Re}}^{ - 1} }}{{\varepsilon^{2} }}\left( {\varepsilon^{2} T_{x}^{2} + T_{y}^{2} } \right) \\ & \quad + \frac{{{\text{Re}}^{ - 1} }}{{\varepsilon^{2} }}\Pr EcA\left[ {\beta + (1 - \beta )\left( {\frac{{\sinh^{ - 1} \left( {C\frac{{{\text{Re}}^{ - 1} }}{\varepsilon }\sqrt {2\varepsilon^{2} v_{y}^{2} + \left( {u_{y} + \varepsilon^{2} v_{x} } \right)^{2} + 2\varepsilon^{2} u_{x}^{2} } } \right)}}{{C\frac{{{\text{Re}}^{ - 1} }}{\varepsilon }\sqrt {2\varepsilon^{2} v_{y}^{2} + \left( {u_{y} + \varepsilon^{2} v_{x} } \right)^{2} + 2\varepsilon^{2} u_{x}^{2} } }}} \right)^{\sigma } } \right]\left\{ \begin{gathered} 2\varepsilon^{2} v_{y}^{2} + 2\varepsilon^{2} u_{x}^{2} \hfill \\ \left( {u_{y} + \varepsilon^{2} v_{x} } \right)^{2} \hfill \\ \end{gathered} \right\}, \\ \end{aligned}$$

where $$\dot{N} = \frac{{T_{\infty }^{2} L^{2} \nu \dot{S}_{gen} }}{{k\left( {T_{w} - T_{\infty } } \right)^{2} U_{\infty } }}$$ and $$A = \frac{{T_{\infty } }}{{T_{w} - T_{\infty } }}$$.

Equation (28) in view of Eqs. (15) and (29) are written without a bar are written as

30 $$\dot{S}_{gen} = \frac{k}{{T_{\infty }^{2} }}\left( {T_{y}^{2} } \right) + \frac{1}{{T_{\infty } }}\left\{ {\mu_{\infty } + \left( {\mu_{o} - \mu_{\infty } } \right)\left( {\frac{{\sinh^{ - 1} \left( {\lambda \sqrt {u_{y}^{2} } } \right)}}{{\lambda \sqrt {u_{y}^{2} } }}} \right)^{\sigma } } \right\}u_{y}^{2} .$$

Now, we apply Eq. (21) in Eq. (30) and get the following form

31 $$\dot{N}_{x} = \underbrace {{\theta^{\prime \prime 2} }}_{{\dot{N}_{x,T} }} + \underbrace {{A\Pr Ec\left\{ {\beta + (1 - \beta )\left( {\frac{{\sinh^{ - 1} \left( {C_{x} \sqrt {F^{\prime \prime 2} } } \right)}}{{C_{x} \sqrt {F^{\prime \prime 2} } }}} \right)^{\sigma } } \right\}F^{\prime \prime 2} }}_{{\dot{N}_{x,v} }},$$

here $$\dot{N}_{x} = \frac{{T_{\infty }^{2} \left( {x + b} \right)^{2} \nu \dot{S}_{gen} }}{{k\left( {T_{w} - T_{\infty } } \right)^{2} u_{w} }}$$.

The average rate of entropy generation is calculated as

32 $$\dot{N}_{av} = \int\limits_{B}^{\infty } {\dot{N}_{x} \left( \eta \right)} d\eta .$$

## Physical parameters

### Skin friction coefficient

To measure the shear stress at the wall, a dimensionless parameter known as the skin friction coefficient is utilized. This coefficient is defined as:

33 $$C_{{f\overline{x}}} = \frac{{\left. {\tau_{12} } \right|_{{\overline{y} = A\left( {\overline{x} + b} \right)^{{\frac{1 - m}{2}}} }} }}{{\frac{1}{2}\rho u_{w}^{2} }}.$$

By using the Eqs. (11) and (21), the coefficient is written as

34 $$C_{fx} = \left. {2{\text{Re}}_{x}^{{ - \frac{1}{2}}} \left\{ {\beta + (1 - \beta )\left( {\frac{{\sinh^{ - 1} \left( {C\sqrt {F^{\prime \prime 2} } } \right)}}{{C\sqrt {F^{\prime \prime 2} } }}} \right)^{\sigma } } \right\}F^{\prime \prime } } \right|_{\eta = B} ,$$

here ${\text{Re}}_{x} = \frac{{u_{w} \left( {x + b} \right)}}{\nu }$ is the local Reynold number.

### Nusselt number

The convective heat transfer coefficient in a dimensionless form which is known Nusselt number, is written as

35 $$Nu_{{\overline{x}}} = - \frac{{k\left. {\frac{{d\overline{T}}}{{d\overline{y}}}} \right|_{{\overline{y} = A\left( {\overline{x} + b} \right)^{{\frac{1 - m}{2}}} }} \left( {\overline{x} + b} \right)}}{{k\left( {T_{w} - T_{\infty } } \right)}}.$$

Equation (35) is written after applying Eqs. (11) and (21) as

36 $$Nu_{x} = - {\text{Re}}_{x}^{{^{\frac{1}{2}} }} \theta^{\prime } \left( B \right).$$

## Solution technique

The numerical result of Eqs. (23) and (24) along Eq. (25) are obtained by using the RK method in the following manner.

Let $F = F_{1}$, $\theta = G_{1}$ and find the system of first-order differential equations as

37 $$\begin{aligned} F_{1}^{\prime } & = F_{2} ,\;F_{2}^{\prime } = F_{3} , \\ F_{3}^{\prime } & = \frac{{\frac{{\left( {m + 1} \right)}}{2}F_{1} F_{3} - mF_{2}^{2} }}{{\left\{ \begin{gathered} \sigma (1 - \beta )\left( {\frac{{\sinh^{ - 1} \left( {C_{x} \sqrt {F_{3}^{2} } } \right)}}{{C_{x} \sqrt {F_{3}^{2} } }}} \right)^{\sigma - 1} \left( { - \frac{{\sinh^{ - 1} \left( {C_{x} \sqrt {F_{3}^{2} } } \right)}}{{C_{x} \sqrt {F_{3}^{2} } }} + \frac{1}{{\sqrt {1 + C_{x}^{2} F_{3}^{2} } }}} \right) \hfill \\ + \left( {\beta + (1 - \beta )\frac{{\sinh^{ - 1} \left( {C_{x} \sqrt {F_{3}^{2} } } \right)}}{{\left( {C_{x} \sqrt {F_{3}^{2} } } \right)}}} \right)^{\sigma } \hfill \\ \end{gathered} \right\}}}, \\ G_{1}^{\prime } & = G_{2} , \\ G_{2}^{\prime } & = - \Pr Ec\left\{ {\beta + (1 - \beta )\left( {\frac{{\sinh^{ - 1} \left( {C_{x} \sqrt {F_{3}^{2} } } \right)}}{{C_{x} \sqrt {F_{3}^{2} } }}} \right)^{\sigma } } \right\}F_{3}^{2} - \frac{{(m + 1)\Pr F_{1} G_{2} }}{2} \\ \end{aligned}$$

along boundary conditions

38 $$\begin{array}{*{20}l} {F_{2} (0) = 1,\;F_{3} (0) = \Omega_{1} } \hfill \\ {G_{1} (0) = 1,\;G_{2} (0) = \Omega_{2} } \hfill \\ \end{array} .$$

Here $\Omega_{1}$ and $\Omega_{2}$ are unknown constants.

## Results and discussion

In this portion, the effects of the time scale parameter $\lambda$, exponent parameter $\sigma$ and high shear rate viscosity $\mu_{\infty }$ on the velocity $F^{\prime } (\eta )$ and temperature $\theta (\eta )$ profiles as well as on skin friction coefficient $C_{fx}$ and Nusselt number, $Nu_{x}$ are examined. The values of dimensionless rheological parameters such as Weissenberg $C$, Prandtl number $\Pr$, Eckert $Ec$, viscosity ratio parameter $\beta$, and Reynold number ${\text{Re}}$ are varied during the investigation and calculated in Table 2.

**Table 2:** The value of different dimensionless parameters when $\rho = 1100$, $C_{p} = 4000$, $\mu_{o} = 0.1$, $m = 3$, $\sigma = 1,$ $a = 0.01$, $A = 0.02$, $b = 0.5$, $T_{w} - T_{\infty } = 30$ and $k = 40$.

$\mathop x\limits_{ \downarrow }$	$\lambda$			*μ*_∞_		
	$\, \mu_{\infty } = 0.03$			$\lambda = 0.7$		
	0.5	0.7	0.9	0.01	0.02	0.03
*C*						
2	2.05	2.87	3.68	2.87	2.87	2.87
3	7.87	11.02	14.16	11.02	11.02	11.02
4	21.50	30.11	38.71	30.11	30.11	30.11
$$10^{ - 6} \times Ec$$						
2	0.02	0.02	0.02	0.02	0.02	0.02
3	1.53	1.53	1.53	1.53	1.53	1.53
4	6.92	6.92	6.92	6.92	6.92	6.92
Re						
2	4297	4297	4297	4297	4297	4297
3	16,507	16,507	16,507	16,507	16,507	16,507
4	45,107	45,107	45,107	45,107	45,107	45,107
Pr						
–	10	10	10	10	10	10
*β*	0.3	0.3	0.3	0.1	0.2	0.3

**Figure 2:**
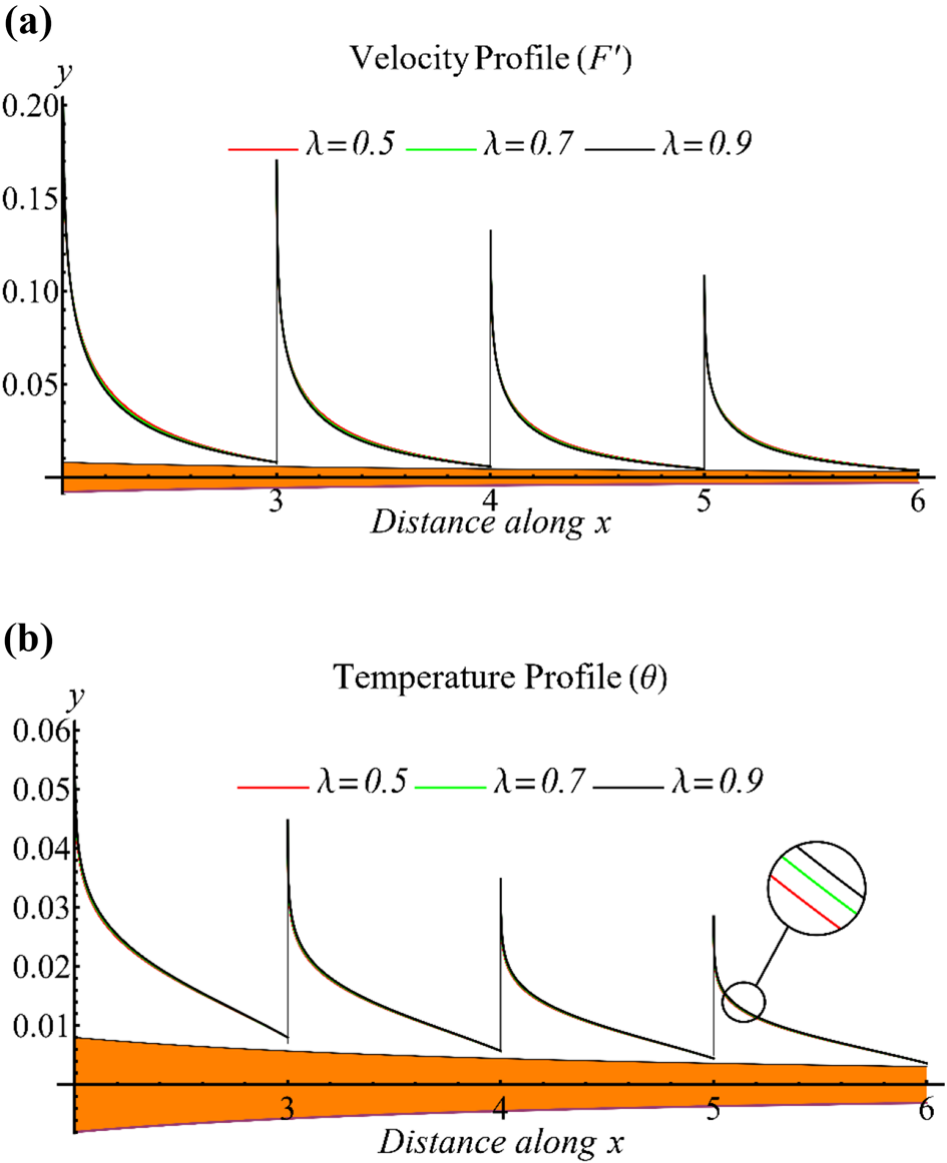
(**a**) The influence of the time sale parameter on the velocity profile. (**b**) The influence of the time sale parameter on the temperature profile.

Figure 2a depicts the velocity profile in terms of the time scale parameter. It is evident that the velocity decreases as the parameter increases. It is used to control the behavior of the viscosity curve. Generally, it is noted that viscosity is rapidly decreased from low shear to high shear when the time scale parameter is increased. The parameter is expressed in the form of the Weissenberg number, indicating a decline in the velocity. Furthermore, the profile's behavior remains almost constant at all values of the $x$, but the velocity boundary layer thickness decreases as the distance from the origin in the parallel direction increases. Figure 2b shows the temperature profile for different time scale parameter values. As the parameter increases, the temperature also rises. Additionally, the temperature distribution remains constant for every value of the $x$, but the thermal boundary layer thickness decreases more than the velocity boundary layer thickness.

**Figure 3:**
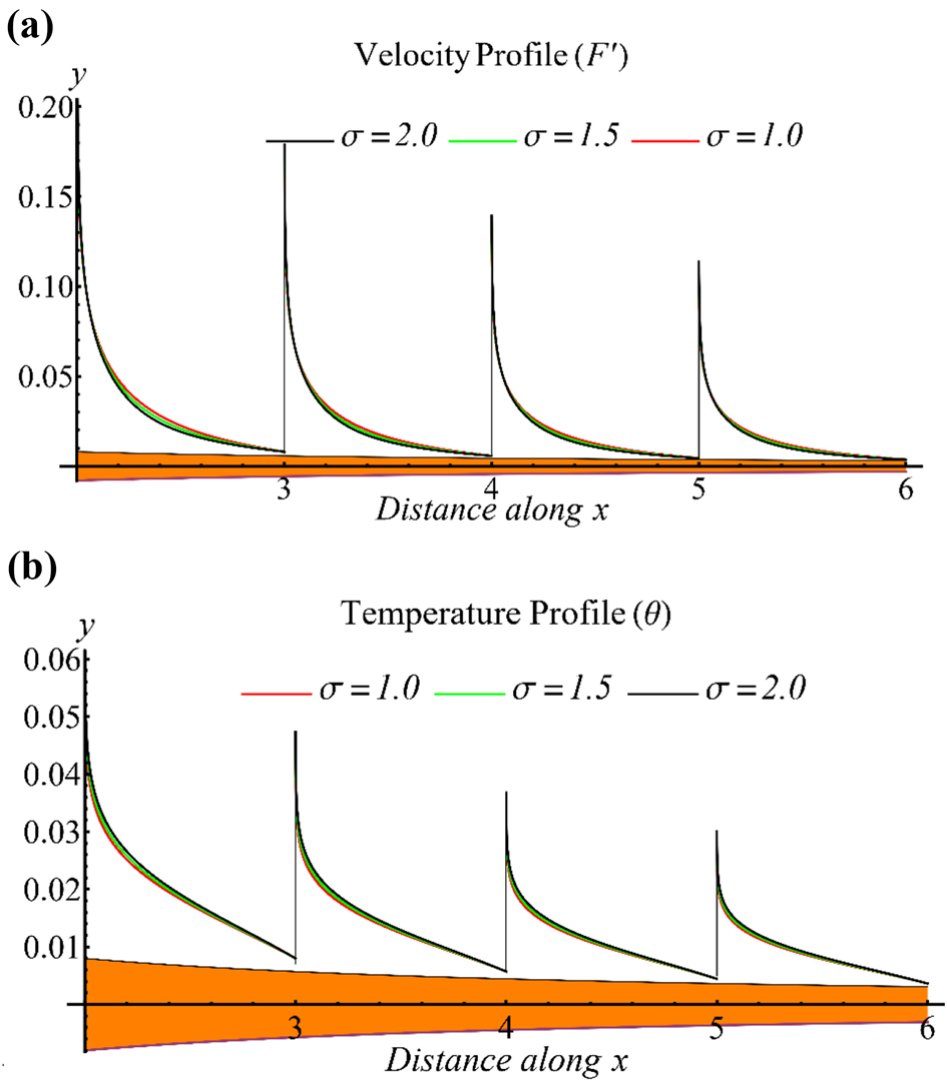
(**a**) The influence of the exponent parameter on the velocity profile. (**b**) The influence of the exponent parameter on the temperature profile.

Figure 3a presents the influence of the exponent parameter on the velocity profile. It is observed that the velocity decreases as the parameter increases, which is attributed to the increase in viscosity with respect to the shear rate under the given flow conditions. Figure 3b depicts the impact of the parameter on the temperature profile, which displays an increase in the temperature profile with an increasing parameter value. Notably, both profiles exhibit a similar trend at different values of $x$, as shown in Figs. 2 and 3.

**Figure 4:**
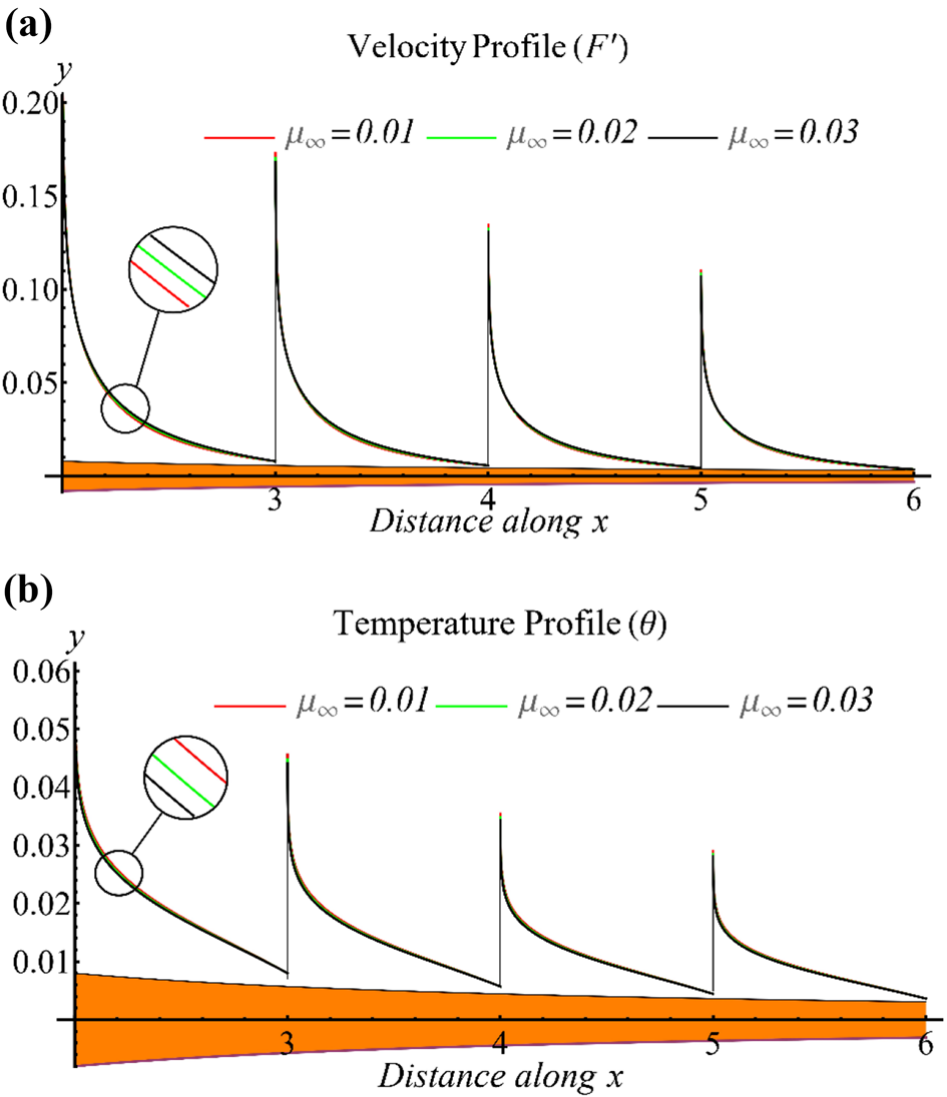
(**a**) The influence of high shear rate viscosity on the velocity profile. (**b**) The influence of high shear rate viscosity on the temperature profile.

Figure 4a reveals the effect of high shear rate viscosity on the velocity profile, which demonstrates an increase in the velocity profile with increasing high shear rate viscosity. This viscosity appears in the viscosity ratio parameter and implies a reduction in velocity as the parameter value increases. Furthermore, Fig. 4b displays the temperature profile under the influence of high shear rate viscosity, indicating a decrease in temperature as the viscosity ratio parameter increases.

**Figure 5:**
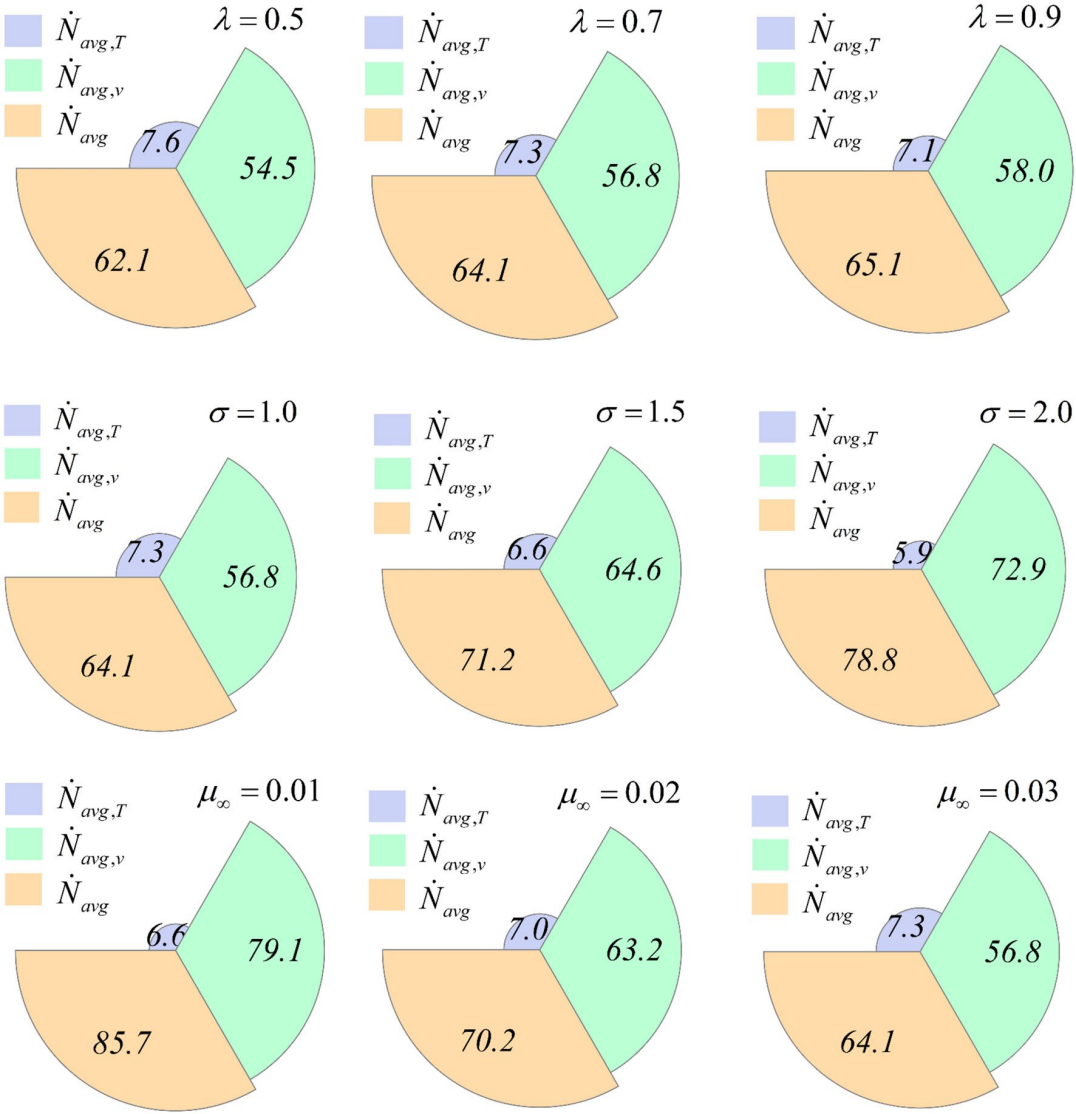
The average rate of entropy generation under different parameter effects.

Figure 5 illustrates the impact of different parameters on the average rate of entropy generation. It is observed that entropy generation increases when the time scale parameter is increased, owing to the dominant influence of viscous irreversibility compared to thermal irreversibility. A similar trend of entropy generation is observed in the case of the exponent parameter, where thermal irreversibility decreases, but viscous irreversibility increases significantly, resulting in an enhancement of entropy generation. When the difference between low and high shear rate viscosities is reduced by increasing the high shear rate viscosity, entropy generation decreases due to the decrease in viscous irreversibility.

The numerical values of the skin friction coefficient under the influence of the time scale parameter, exponent parameter, and high shear rate viscosity at different values of the parameter are presented in Table 3. The coefficient magnitude decreases with increasing time scale and exponent parameters but increases with an increase in high shear rate viscosity. The negative sign of the coefficient indicates that the sheet exerts a shear stress on the fluid. The results of the Nusselt number in relation to the aforementioned parameters are presented in Table 4. An increase in the time scale and exponent parameters results in a decrease in the Nusselt number, while high shear rate viscosity leads to an increase in the Nusselt number.

**Table 3:** The values of local Skin friction number $- C_{{f_{x} }}$ under the influence of different parameters.

	$\lambda$			$\sigma$			$\mu_{\infty }$		
$\mathop x\limits_{ \downarrow }$	0.5	0.7	0.9	0.010	0.015	0.020	0.01	0.02	0.03
2	0.04005	0.03623	0.03364	0.03786	0.03204	0.02572	0.03390	0.03897	0.04404
3	0.01399	0.01296	0.01233	0.01362	0.00993	0.00699	0.00921	0.01270	0.01618
4	0.00695	0.00663	0.00644	0.00701	0.00481	0.00323	0.00388	0.00618	0.00847

**Table 4:** The values of local Nusselt number $- Nu_{x}$ under the influence of different parameters.

	$\lambda$			$\sigma$			$\mu_{\infty }$		
$\mathop x\limits_{ \downarrow }$	1.0	1.5	2.0	0.010	0.015	0.020	0.01	0.02	0.03
2	125.143	385.727	370.563	123.398	119.052	114.371	118.945	121.491	123.398
3	245.281	241.860	239.285	241.860	233.341	224.168	233.133	238.123	241.860
4	405.464	399.810	395.553	399.810	385.727	370.563	385.383	393.632	399.810

## Conclusions

The present investigation examines the heat and mass flow behaviors of a shear-thinning fluid through a generalized Powell–Eyring model. This study introduces a higher shear rate viscosity, time scale and exponent parameters in the flow modeling, which has not been previously done with the Powell–Eyring model. The effects of these parameters on the velocity and temperature profiles are summarized as follows:

- Upon increasing the time scale parameter, a consequential reduction in viscosity occurs, leading to a decrease in the velocity profile and an increase in the temperature profile. The corresponding increase in the time scale parameter leads to an increase in the average entropy generation.
- An increase in the exponent parameter accentuates the shear thinning effects, resulting in a decrease in the velocity profile and an increase in the temperature profile. This parameter also causes an increase in the average entropy generation.
- Regarding the numerical values, an increase in the time scale and exponent parameters leads to a decrease in the skin friction coefficient. However, an increase in shear rate viscosity causes the skin friction coefficient to increase. Conversely, the Nusselt number demonstrates an opposing trend in comparison to the skin friction coefficient concerning these parameters.

## Data Availability

The datasets used and/or analyzed during the current study are available from the corresponding author upon request.
